# Evaluation of efficacy and safety of pirfenidone 200 mg tablets in patients with idiopathic pulmonary fibrosis in a real-life setting

**DOI:** 10.3906/sag-2102-262

**Published:** 2021-09-25

**Authors:** Funda COŞKUN, Aykut ÇİLLİ, İsmail HANTA, Can SEVİNÇ, Ayşe ÖDEMİŞ, Ahmet URSAVAŞ

**Affiliations:** 1 Department of Chest Diseases, Faculty of Medicine, Bursa Uludağ University, Bursa, Turkey; 2 Department of Chest Diseases, Faculty of Medicine, Akdeniz University, Antalya, Turkey; 3 Department of Chest Diseases, Faculty of Medicine, Çukurova University, Adana, Turkey; 4 Department of Chest Diseases, Faculty of Medicine, Dokuz Eylül University, Izmir, Turkey

**Keywords:** Cough, idiopathic pulmonary fibrosis, treatment

## Abstract

**Background/aim:**

Phase III trials have demonstrated a significant efficacy and an acceptable safety for pirfenidone in patients having mild to moderate idiopathic pulmonary fibrosis (IPF). Real-life data on the use of pirfenidone 200 mg tablets are limited. This study aimed to investigate the efficacy and safety of pirfenidone 200 mg tablets for the treatment of IPF in a real-life setting.

**Materials and methods:**

A retrospective, multicenter study conducted in four university hospitals in Turkey between January 2017 and January 2019. Clinical records of patients diagnosed with mild to moderate IPF and receiving pirfenidone (200 mg tablets, total 2400 mg/day) were reviewed retrospectively and consecutively. Pulmonary function measurements including forced vital capacity (FVC%) and diffusing capacity of the lungs for carbon monoxide (DLCO%) were analyzed at baseline and after 6-month of pirfenidone treatment. Descriptive statistics were expressed as mean, standard error or median (minimum-maximum), number and percentage, where appropriate.

**Results:**

The study included 82 patients, of whom 87.8% were males (mean age, 66 years). After 6-month of treatment, 7 patients discontinued the treatment. Of the remaining 75 patients, 71 (94.6%) remained stable, 4 (5.4%) had progressive disease as evident by a decline in the FVC% of at least 10% while on treatment, and 45 (61.3%) had improved cough. At least one adverse event (AE) associated with the treatment was observed in 28 (37.3%) patients.

**Conclusion:**

Pirfenidone 200 mg was effective and well tolerated and associated with relatively mild and manageable AEs in IPF patients.

## 1. Introduction

Idiopathic pulmonary fibrosis (IPF) is a fatal condition characterized by chronic progressive lung fibrosis [1–3]. Patients with IPF have a 5-year survival rate of 20%–40% [2,4]. Nevertheless, introduction of novel antifibrotic therapies has prolonged the average life expectancy of these patients to 9.9 years [3]. Although no cure exists for IPF, it is possible to slow down the progression of the disease and to reduce its exacerbations [5–9]. Currently, only two drugs, which are nintedanib and pirfenidone, are available on the market for the treatment of IPF.

In Turkey, two strengths (200 mg and 267 mg) of pirfenidone are available in the market. No restrictions are applied to the selection of the molecule or formulation by the Turkey health authority. A physician is free to choose the appropriate treatment based on the individual patient’s conditions, ease-of-use, side-effect profile, and dosing regimen. The target in the treatment is to titrate the dose to a maximum of 2400 mg/day or 2403 mg/day.

Pirfenidone was first used in some studies conducted in Japan, and patients with IPF were treated with 200 mg tablets to a maximum target dose of 1800 mg/day or 2400 mg/day [10,11]. These studies have demonstrated that the use of pirfenidone slows down the decline of forced vital capacity (FVC) and is associated with a reduction in exacerbations. In two major double-blind randomized controlled trials (the ASCEND trial conducted in Australia, Brazil, Croatia, Israel, Mexico, New Zealand, Peru, Singapore, and the United States and the CAPACITY trial conducted in Australia, Europe, and North America), patients with IPF were randomly assigned to receive either capsules containing 267 mg of pirfenidone (a total dose of 2403 mg/day) or to placebo [12,13]. In these trials, pirfenidone was demonstrated to reduce disease progression, improve progression-free survival, and decrease mortality [12,13].

To the best of our knowledge, no data is currently available for the use of pirfenidone with its 200 mg dose formulation in IPF patients outside of Japan. Accordingly, the present retrospective study aimed to investigate the efficacy and safety of pirfenidone 200 mg tablets titrated to the target total dose (2400 mg/day) in patients with IPF in four university hospitals of Turkey.

## 2. Materials and methods

Clinical records of patients diagnosed with mild to moderate IPF and receiving pirfenidone treatment (pirfenidone 200 mg tablets, total daily dose of 2400 mg) in four university hospitals (Akdeniz University, Bursa Uludağ University, Çukurova University, and Dokuz Eylül University) in Turkey between January 2017 and January 2019 were reviewed retrospectively and consecutively. The present study was approved by the Clinical Research Ethics Committee of Bursa Uludağ University Medical Faculty (approval number: 2018-7/28, dated: 10 April 2018). The study was carried out in compliance with the Declaration of Helsinki and in accordance with the Good Clinical Practice (GCP) guidelines. Informed consents of all participants were obtained.

The diagnosis of IPF was established based on the 2018 American Thoracic Society (ATS)/the European Respiratory Society (ERS)/the Japanese Respiratory Society (JRS)/the Latin American Thoracic Association (ALAT) criteria [7]. Pulmonary function measurements including percent predicted FVC (FVC%) and percentage of diffusing capacity of the lungs for carbon monoxide (DLCO%) were analyzed at baseline and after the patients received pirfenidone for at least 6 months.

Disease severity was rated using the Gender-Age-Physiology (GAP) index [8]. In the GAP index, the patients are grouped according to the variable categories (sex, age, and physiologic variables [FVC% and DLCO%]), and the relevant points are assigned for each group as follows: 1) sex: females (0 point) and males (1 point), 2) age: ≤ 60 years (0 point), 61 – 65 years (1 point), > 65 years (2 points); 3) physiologic variables: a) FVC%: ≥ 75% (0 point), 50%–75% (1 point), and < 50% (2 points) and b) DLCO%: > 55% (0 point), 36%–55% (1 point), and < 35% (2 points). Patients who are unable to perform the diffusing capacity test are assigned 3 points. A total GAP score of 0–3 indicates a mild disease, a total GAP score of 4–5 indicates intermediate disease, and a total GAP score of 6–8 indicates a severe disease.

The patients were followed at monthly intervals for the first 6 months and then quarterly if their condition was stable under treatment. IPF was defined as stable in case of no change and/or a decrease of < 10% in the FVC%. IPF was defined as progressed in the presence of a decrease by ≥ 10% in the FVC%. Each clinical review included confirmation and documentation of treatment dose, presence of any new adverse events (AEs), strategies for the management of AEs, documentation of dose interruptions, and hospital admissions in the preceding months. Weight loss was defined as a reduction in body weight by > 10% from baseline within 6 months. Cough was defined as “worse”, “better”, or “unchanged” based on patients’ self-report.

### 2.1. Statistical analysis

Statistical analyses were performed using the IBM SPSS Statistics for Windows, Version 22.0 (IBM Corp., Armonk, NY, USA). Descriptive statistics were expressed as mean, standard error, or median (minimum-maximum) for continuous variables and number (n) and percentage (%) for categorical variables.

## 3. Results

The present study included 82 patients with IPF, of whom 87.8% were males with a mean age of 66 ± 1.2 years (range, 44 – 85 years). The demographic and clinical characteristics of the patients with IPF are summarized in Table 1. Sixty-two (75.6%) patients had a definite usual interstitial pneumonia (UIP) pattern on high-resolution computed tomography (HRCT) scans, whereas 18 (22%) patients had surgically proven UIP patterns. The mean GAP index score in the whole study group was 3.76 ± 1.45. Accordingly, the severity of IPF among the study population was distributed as mild to moderate.

The findings of the respiratory function tests in the study population are summarized in Table 2. After 6 months of treatment, of 82 patients with IPF, 7 patients discontinued the treatment due to AEs; of the remaining 75 patients, 71 (94.6%) remained stable and 4 (5.4%) had progressive disease as evident by a decline in the FVC% by at least 10% while on treatment (Figure), and 45 (61.3%) had an improvement in cough.

Seven (8.5%) out of 82 IPF patients discontinued pirfenidone due to AEs; discontinuation of treatment was due to severe photosensitivity in 2 patients and due to gastrointestinal complaints in 5 patients. Of the remaining 75 patients continued to treatment, 28 (37.3%) had at least one AE associated with the treatment. The most frequently reported AE was gastrointestinal discomfort (n = 15; 20%), followed by weight loss (n = 11; 14%), rash/itching (n = 5; 6.7%), and photosensitivity (n = 5; 6.7%). For the management of AEs, the patients were informed about taking medicines with food to reduce gastrointestinal complaints, those with weight loss were recommended a high calorie diet at frequent intervals, and those with rash/itching and photosensitivity were informed about sun protection measures and recommended sunscreen creams and therapeutic local treatments for the skin. Dose adjustment was required in 10 (12%) patients who experienced AEs (Table 3). In these 10 patients, pirfenidone was adjusted to a dose of 1800 mg/day. Additionally, the average dose of pirfenidone was 2311 mg/day in the patients who completed their treatment. The time to treatment discontinuation due to AEs varied between 1 month and 3 months. During the treatment period, exacerbation was observed in 2 (2.7%) patients.

## 4. Discussion

The present retrospective study revealed that the use of pirfenidone 200 mg tablets for 6 months in IPF patients was both effective and well-tolerated. Although the study was not placebo-controlled, it was observed that the FVC% value in the study patients remained stable at 94.6% at the end of the 6-month treatment. Weight loss was observed in 14% of the patients.

Pirfenidone has been used in Japan for the treatment of IPF since 2000s. Pirfenidone has been shown to be effective in reducing the decline in FVC value and improving the 6-min walking distance [12,13]. Pirfenidone is believed to exert its effect through transforming growth factor-beta (TGF-β) [4–6]. In their study, Azuma et al. [10] demonstrated that as compared with placebo, pirfenidone was associated with a reduction in FVC decline and provided protection against exacerbations during a 9-month follow-up period. Based on its favorable efficacy and safety profile, pirfenidone is one of the two drugs approved for IPF treatment by the US Food and Drug Administration (FDA) and the European Medicines Agency (EMA). Currently, in our country, two strengths (267 mg and 200 mg) of pirfenidone are available in the market. Patients who were titrated to the optimal dose with pirfenidone 200 mg tablets were included in the present study.

In two randomized controlled trials on the efficacy of pirfenidone in IPF patients, the FVC value was used as a primary endpoint [12,13]. In the CAPACITY trial, the reduction in decline in FVC at the end of 72 weeks was reported to be significant in the patient group than in the placebo group [12]. In the post-hoc analysis of the placebo-controlled ASCEND (study 016; NCT01366209) and CAPACITY (studies 004 and 006; NCT00287716 and NCT00287729) phase III trials, Nathan et al. [14] observed a significant difference in the declines in FVC% in favor of the pirfenidone group. In the present study, the efficacy of pirfenidone treatment was also evaluated based on FVC values. Of the patients continued on pirfenidone treatment at the end of 6 months, 94.6% had a decline in the FVC% by <10% and were considered to have stable disease. This finding was consistent with those in the literature. On the other hand, in a long-term study on the outcomes of IPF patients (n = 502) who received pirfenidone for 3 years, Bando et al. [15] demonstrated that FVC was preserved in more than 50% of their patients. Accordingly, one of the limitations of the present study could be considered its short evaluation period (6 months), and, thus, it should be kept in mind that this study only provided limited information on disease progression.

Cough is a major clinical problem among IPF patients. The underlying mechanism is not clear and patients do not respond to antitussive therapy [16]. In their study, Van Manen et al. [17] reported a reduction in 24-h cough counts by 34% after 12 weeks of pirfenidone treatment in IPF patients. In the present study, 61.3% of the patients had an improvement in cough after 6 months of treatment. Although an objective scale for evaluating cough severity was not used, which can be considered one of the limitations of the present study, the observed improvement in cough in 61.3% of the patients was considered important as the present study reflects the situation in a real-life setting. During the study period, cough was evaluated based on patients’ self-report as “worse”, “better”, or “unchanged”. An important finding in the present study was that a reduction in cough frequency was demonstrated after 6 months of pirfenidone treatment in a real-life setting.

In their studies, Arai et al. [18] and Hanta et al. [19] demonstrated a better response to pirfenidone treatment in mild cases of IPF. Using the criteria of the Japanese Respiratory Society, the severity of the disease is classified based on oxygen saturation at rest and on arterial oxygen pressure [20]. Another study conducted in Japan showed that pirfenidone had similar effects in patients receiving a total daily dose of 1200 mg and in those receiving a total daily dose of 1800 mg using 200 mg tablets [11]. In their phase III trial, Taguchi et al. [21] demonstrated a reduction in FVC decline with pirfenidone in all grades of disease severity and observed a better response in mild to moderate IPF patients. Although these results have suggested that initiation to pirfenidone at an early stage of IPF might be associated with a better response, the study by Okuda et al. [22] provided evidence that pirfenidone was also effective in severe IPF patients. The above-mentioned studies were all conducted in Japan using the 200 mg strength of pirfenidone. In the present study, oxygen saturation was not used as a measure of severity; however, the GAP index was used for the assessment of disease severity [8]. Based on their GAP scores, the present study population can be considered to have mild to moderate disease. By the end of the 6-month study period, a pirfenidone response similar to the Japanese trials was demonstrated.

In many studies, the most common AE associated with pirfenidone is gastrointestinal discomfort [12,13,23]. Nausea and vomiting and-although relatively infrequent-anorexia have been reported. In the present study, at least one AE associated with pirfenidone treatment was observed in 28 (37.3%) patients. Gastrointestinal discomfort (20%), weight loss (14%), rash/itching (6.6%), and photosensitivity (6.6%) were the most frequent AEs in our cohort. A comprehensive safety outcome analysis of pirfenidone conducted in a large and well-defined IPF cohort (n = 1299), which had prospective follow-up period of 9.9 years, pirfenidone was reported to be safe and usually well tolerated [23]. Gastrointestinal system and skin-related events were reported as the most common AEs related to pirfenidone, the severity of which were usually mild to moderate and which improved with dose modification [23]. In that particular study, increased aminotransferase levels, which were transient and reversible with no clinical sequelae upon dose modification or treatment discontinuation, were also reported mostly in the first 6 months of treatment with pirfenidone [23]. Taking the evaluation period of the present study (6 months) into account, none of our patients had elevated aminotransferase levels. Another AE associated with the use of antifibrotic agents is weight loss [24]. Recent studies have reported weight loss in patients on pirfenidone or nintedanib. In their study investigating weight loss in IPF patients (n = 80) using pirfenidone or nintedanib, Perelas et al. [24] reported a 5% weight-loss in 44% of their patients and a 10% weight loss in 19% of their patients. They also reported a significantly more frequent weight loss in the nintedanib group than in the pirfenidone group [24]. In the present study, weight loss was considered an AE during data collection and the findings were found to be consistent with those reported in the literature. Overall, the observed AE profile in our cohort was consistent with those reported in the literature, and no unusual AEs were observed.

In the present study, 71% of the patients remained on pirfenidone treatment for 6 months without a need to modify the dose; dose adjustment was required in 10 (12%) patients. Similarly, in a long-term study in which patients were divided into those who used pirfenidone for less than 1 year or more than 1 year, Ogawa et al. [25] reported that more than 65% of their patients remained on pirfenidone therapy for 1 year or longer and that long-term use of pirfenidone was effective and safe.

Except for photosensitivity, most AEs usually disappear after 3 months on treatment. As was demonstrated in the present study and reported in the literature, most AEs are mild to moderate as they usually disappear with tapering off or discontinuation of treatment. In phase III trials on pirfenidone, the rate of tapering off or discontinuation of treatment was reported as 46% and 41%, respectively [13,14]. In the ASCEND trial, the observed AEs resulted in discontinuation of treatment in 40 (14.4%) patients in the pirfenidone group and in 30 (10.8%) patients in the placebo group [13]. The most common AE that resulted in treatment discontinuation was reported as worsening of IPF in 3 (1.1%) patients in the pirfenidone group and in 15 (5.4%) patients in the placebo group [13]. Other AEs leading to treatment discontinuation in at least 1% of the patients in the pirfenidone group included elevated liver enzymes, pneumonia, rash, and weight loss, with each occurring in 3 patients (1.1%) [13]. Following the approval of pirfenidone in Japan in 2008, Ogura et al. [26] conducted a post-marketing surveillance on 1371 patients. At the end of a long follow-up over 12 months, 48.7% of the patients were still on treatment, and the most common reason for treatment discontinuation was AEs reported in 24.3% of the patients. In the study on the outcomes of IPF patients (n = 502) who received pirfenidone for 3 years by Bando et al. [15], 37.1% of the patients discontinued treatment in less than 1 year due to AEs and the 3-year incidence of all AEs was reported as 32.6%. In the present study, 8.5% of the patients discontinued treatment due to AEs at the end of the 6-month follow-up and the rate of AEs was 37.3%. Although AEs are most frequently reported in the first 6 months of pirfenidone treatment, treatment discontinuation due to AEs was uncommon in the present study. This may be partly due to the short evaluation period (6 months) of the study. Considering the relatively short follow-up period, the treatment discontinuation rate found in the present study appeared to be comparable.

In addition to the limitations mentioned above, some other limitations of the present study included its retrospective design, small sample size, and assessment of cough based on patients’ self-reports. Nevertheless, to the best of our knowledge, this study was the first to report efficacy and safety of 200 mg pirfenidone tablets titrated to the target total dose in patients from four different regions of Turkey; that is, outside of Japan, in a real-life setting.

In conclusion, the findings of the present study demonstrated that FVC could be preserved for a period of 6 months in IPF patients who were titrated to the target dose using 200 mg tablets of pirfenidone and that the associated AEs were mild to moderate in severity and were manageable. Treating IPF patients with 200 mg pirfenidone tablets to the target dose could be suggested as both efficacious and safe.

## Figures and Tables

**Figure:**
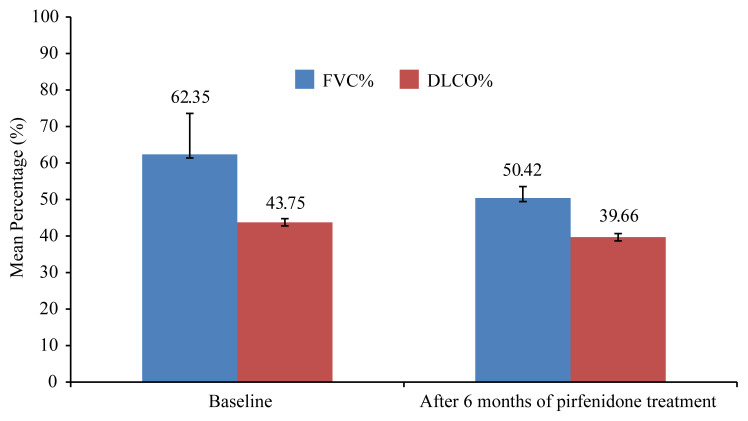
Changes in forced vital capacity (FVC%) and diffusing capacity of the lungs for carbon monoxide (DLCO%) after 6-month of pirfenidone treatment according to the baseline in four patients having progressive disease.

**Table 1:** Demographics and clinical characteristics of the patients with idiopathic pulmonary fibrosis at baseline.

Characteristics	Patients with IPF n = 82
Age, years, Mean ± SE, Min - Max	61.27 ± 8.38 44 – 85
Sex, Male/Female	72/10
BMI, kg/m^2^, Mean ± SE	28.3 ± 3.7
Smoking status, n (%)	
Current smokers	2 (2.4)
Quitters	58 (70.7)
Never smokers	22 (26.8)
HRCT, n (%)	
Definite UIP	62 (75.6)
Probable	11 (13.4)
Indeterminate	5 (6.1)
Alternative	4 (4.9)
Biopsy-proven UIP pattern, n (%)	
Yes	18 (22.0)
No	64 (78.0)
Comorbidities as defined by the physician, n (%)	
Atherosclerotic heart disease	10 (12.2)
Hypertension	28 (34.2)
Diabetes mellitus	22 (26.8)
Arrhythmia	4 (4.9)
GAP index score, Mean ± SE	3.76 ± 1.45

IPF, idiopathic pulmonary fibrosis; Min-Max, minimum-maximum; SE, standard error; BMI, body mass index; HRCT, high-resolution computed tomography; UIP, usual interstitial pneumonia.

**Table 2:** Findings of the respiratory function tests in the study patients.

	Baseline Mean ± SE (n = 82)	At the end of 6^th^ month Mean ± SE (n = 75)
FVC, % predicted	69.44 ± 17.04	71.42 ± 18.31
FEV_1_/FVC	90.56 ± 6.62	89.80 ± 6.05
DLCO_adj_	52.46 ± 16.74	55.43 ± 15.13

SE, standard error; FVC, forced vital capacity; FEV_1_, forced expiratory volume in 1 second; DLCO_adj_, adjusted diffusing capacity of the lungs for carbon monoxide.

**Table 3:** Adherence to pirfenidone treatment in the study patients

Actions taken	Patients with IPF (n = 82) n (%)
No dose modification	58 (71)
Reduction in pirfenidone dose	10 (12)
Temporary discontinuation of pirfenidone	7 (8.5)
Permanent discontinuation of pirfenidone	7 (8.5)

IPF, idiopathic pulmonary fibrosis.

## References

[1] Raghu G Collard HR Egan JJ Martinez FJ Behr J An official ATS/ERS/JRS/ALAT statement: idiopathic pulmonary fibrosis: evidence-based guidelines for diagnosis and management American Journal of Respiratory Critical Care Medicine 2011 183 788 824 10.1164/rccm.2009-040GL 21471066 PMC5450933

[2] Fernández Pérez ER Daniels CE Schroeder DR St Sauver J Hartman TE Incidence, prevalence, and clinical course of idiopathic pulmonary fibrosis: a population-based study Chest 2010 137 129 137 10.1378/chest.09-1002 19749005 PMC2803118

[3] Costabel U Albera C Bradford WZ Hormel P King TE Jr Analysis of lung function and survival in RECAP: An open-label extension study of pirfenidone in patients with idiopathic pulmonary fibrosis Sarcoidosis Vascular Diffuse Lung Disease 2014 31 198 205 25363219

[4] Meltzer EB Noble PW Idiopathic pulmonary fibrosis Orphanet Journal of Rare Diseases 2008 3 8 10.1186/1750-1172-3-8 18366757 PMC2330030

[5] Collard HR King TE Jr Bartelson BB Vourlekis JS Schwarz MI Changes in clinical and physiologic variables predict survival in idiopathic pulmonary fibrosis American Journal of Respiratory Critical Care Medicine 2003 168 538 542 10.1164/rccm.200211-1311OC 12773325

[6] Kim DS Collard HR King TE Jr Classification and natural history of the idiopathic interstitial pneumonias Proceedings of American Thoracic Society 2006 3 285 292 10.1513/pats.200601-005TK PMC2658683 16738191

[7] Raghu G Remy-Jardin M Myers JL Richeldi L Ryerson CJ Diagnosis of idiopathic pulmonary fibrosis. An official ATS/ERS/JRS/ALAT clinical practice guideline American Journal of Respiratory Critical Care Medicine 2018 198 e44 e68 10.1164/rccm.201807-1255ST 30168753

[8] Ley B Ryerson CJ Vittinghoff E Ryu JH Tomassetti S A multidimensional index and staging system for idiopathic pulmonary fibrosis Annals of Internal Medicine 2012 156 684 691 10.7326/0003-4819-156-10-201205150-00004 22586007

[9] Hunninghake GW Kalica AR Approaches to the treatment of pulmonary fibrosis American Journal of Respiratory Critical Care Medicine 1995 151 3 915 918 10.1164/ajrccm.151.3.7881692 7881692

[10] Azuma A Nukiwa T Tsuboi E Suga M Abe S Double-blind, placebo-controlled trial of pirfenidone in patients with idiopathic pulmonary fibrosis American Journal of Respiratory Critical Care Medicine 2005 171 1040 1047 10.1164/rccm.200404-571OC 15665326

[11] Taniguchi H Ebina M Kondoh Y Ogura T Azuma A Pirfenidone in idiopathic pulmonary fibrosis European Respiratory Journal 2010 35 821 829 10.1183/09031936.00005209 19996196

[12] Noble PW Albera C Bradford WZ Costabel U Glassberg MK Pirfenidone in patients with idiopathic pulmonary fibrosis (CAPACITY): two randomised trials Lancet 2011 377 1760 1769 10.1016/S0140-6736(11)60405-4 21571362

[13] King TE Jr Bradford WZ Castro-Bernardini S Fagan EA Glaspole I A phase 3 trial of pirfenidone in patients with idiopathic pulmonary fibrosis New England Journal of Medicine 2014 370 2083 2092 10.1056/NEJMoa1402582 24836312

[14] Nathan SD Costabel U Glaspole I Glassberg MK Lancaster LH Efficacy of pirfenidone in the context of multiple disease progression events in patients with idiopathic pulmonary fibrosis Chest 2019 155 712 719 10.1016/j.chest.2018.11.008 30472023

[15] Bando M Yamauchi H Ogura T Taniguchi H Watanabe K Japan Pirfenidone Clinical Study Group Clinical experience of the long-term use of pirfenidone for idiopathic pulmonary fibrosis Internal Medicine 2016 55 443 448 10.2169/internalmedicine.55.5272 26935361

[16] van Manen MJG Wijsenbeek MS Cough, an unresolved problem in interstitial lung diseases Current Opinion Supportive Palliative Care 2019 13 143 151 10.1097/SPC.0000000000000447 31365459

[17] van Manen MJG Birring SS Vancheri C Vindigni V Renzoni E Effect of pirfenidone on cough in patients with idiopathic pulmonary fibrosis European Respiratory Journal 2017 50 1701157 10.1183/13993003.01157-2017 29051272 PMC5678896

[18] Arai T Inoue Y Sasaki Y Tachibana K Nakao K Predictors of the clinical effects of pirfenidone on idiopathic pulmonary fibrosis Respiratory Investigation 2014 52 136 143 10.1016/j.resinv.2013.09.002 24636270

[19] Hanta I Cilli A Sevinc C The effectiveness, safety, and tolerability of pirfenidone in idiopathic pulmonary fibrosis: A retrospective study Advances in Therapy 2019 36 1126 1131 10.1007/s12325-019-00928-3 30900199

[20] Homma S Sugino K Sakamoto S Usefulness of a disease severity staging classification system for IPF in Japan: 20 years of experience from empirical evidence to randomized control trial enrollment Respiratory Investigation 2015 53 7 12 10.1016/j.resinv.2014.08.003 25542598

[21] Taguchi Y Ebina M Hashimoto S Ogura T Azuma A Efficacy of pirfenidone and disease severity of idiopathic pulmonary fibrosis: Extended analysis of phase III trial in Japan Respiratory Investigation 2015 53 279 287 10.1016/j.resinv.2015.06.002 26521105

[22] Okuda R Hagiwara E Baba T Kitamura H Kato T Safety and efficacy of pirfenidone in idiopathic pulmonary fibrosis in clinical practice Respiratory Medicine 2013 107 1431 1437 10.1016/j.rmed.2013.06.011 23849626

[23] Lancaster L Albera C Bradford WZ Costabel U du Bois RM Safety of pirfenidone in patients with idiopathic pulmonary fibrosis: integrated analysis of cumulative data from 5 clinical trials British Medical Journal Open Respiratory Research 2016 3 e000105 10.1136/bmjresp-2015-000105 PMC4716177 26835133

[24] Perelas A Glennie J van Kerkhove K Li M Scheraga RG Choice of antifibrotic medication and disease severity predict weight loss in idiopathic pulmonary fibrosis Pulmonary Pharmacology and Therapeutics 2019 59 101839 10.1016/j.pupt.2019.101839 31518649 PMC6889056

[25] Ogawa K Miyamoto A Hanada S Takahashi Y Murase K The efficacy and safety of long-term pirfenidone therapy in patients with idiopathic pulmonary fibrosis Internal Medicine 2018 57 2813 2818 10.2169/internalmedicine.0559-17 29780123 PMC6207833

[26] Ogura T Azuma A Inoue Y Taniguchi H Chida K All-case post-marketing surveillance of 1371 patients treated with pirfenidone for idiopathic pulmonary fibrosis Respiratory Investigation 2015 53 232 241 10.1016/j.resinv.2015.06.001 26344613

